# Rule based classifier for the analysis of gene-gene and gene-environment interactions in genetic association studies

**DOI:** 10.1186/1756-0381-4-4

**Published:** 2011-03-01

**Authors:** Thorsten Lehr, Jing Yuan, Dirk Zeumer, Supriya Jayadev, Marylyn D Ritchie

**Affiliations:** 1 Boehringer Ingelheim Pharma GmbH & Co. KG, Department of Drug Metabolism and Pharmacokinetics, 88397 Biberach an der Riss, Germany; 2 Boehringer Ingelheim Pharma Inc., Department of Toxicology and Safety Assessment, 900 Ridgebury Road, Ridgefield, CT 06877, USA; 3 Boehringer Ingelheim Pharma Inc., Department of Research Operations, 900 Ridgebury Road, Ridgefield, CT 06877, USA; 4 Vanderbilt University Medical Center, Department of Molecular Physiology & Biophysics, Center for Human Genetics Research, 519 Light Hall, Nashville, TN 37232, USA

## Abstract

**Background:**

Several methods have been presented for the analysis of complex interactions between genetic polymorphisms and/or environmental factors. Despite the available methods, there is still a need for alternative methods, because no single method will perform well in all scenarios. The aim of this work was to evaluate the performance of three selected rule based classifier algorithms, RIPPER, RIDOR and PART, for the analysis of genetic association studies.

**Methods:**

Overall, 42 datasets were simulated with three different case-control models, a varying number of subjects (300, 600), SNPs (500, 1500, 3000) and noise (5%, 10%, 20%). The algorithms were applied to each of the datasets with a set of algorithm-specific settings. Results were further investigated with respect to a) the Model, b) the Rules, and c) the Attribute level. Data analysis was performed using WEKA, SAS and PERL.

**Results:**

The RIPPER algorithm discovered the true case-control model at least once in >33% of the datasets. The RIDOR and PART algorithm performed poorly for model detection. The RIPPER, RIDOR and PART algorithm discovered the true case-control rules in more than 83%, 83% and 44% of the datasets, respectively. All three algorithms were able to detect the attributes utilized in the respective case-control models in most datasets.

**Conclusions:**

The current analyses substantiate the utility of rule based classifiers such as RIPPER, RIDOR and PART for the detection of gene-gene/gene-environment interactions in genetic association studies. These classifiers could provide a valuable new method, complementing existing approaches, in the analysis of genetic association studies. The methods provide an advantage in being able to handle both categorical and continuous variable types. Further, because the outputs of the analyses are easy to interpret, the rule based classifier approach could quickly generate testable hypotheses for additional evaluation. Since the algorithms are computationally inexpensive, they may serve as valuable tools for preselection of attributes to be used in more complex, computationally intensive approaches. Whether used in isolation or in conjunction with other tools, rule based classifiers are an important addition to the armamentarium of tools available for analyses of complex genetic association studies.

## Background

Genetic association studies aim to identify the contribution of genetic polymorphisms to specific phenotypes such as disease status, drug responder status, and adverse drug reactions [1]. Association studies have been gaining interest as genotyping costs have significantly decreased and as more association-based success stories are reported [2,3].

In addition to the impact of a single genetic locus, more complex interactions between genetic polymorphisms and/or environmental factors, such as age, weight, and drug exposure can provide more accurate models for the prediction of complex phenotypes [4]. Several methods are currently available for the analysis of gene-gene and gene-environment interactions, e.g. random forests, focused interaction testing frameworks, stepwise logistic regression, explicit logistic regression, Multifactor Dimensionality Reduction (MDR) and Neural Networks (NN) [5,6].

One of the most frequently used algorithm, MDR, allows an exhaustive search for complex interactions [7]. MDR has a good sensitivity to detect gene-gene and gene-environment interactions; however, the algorithm faces two challenges. First, large computational resources are required to perform the exhaustive searching, and the search for higher interaction models can be time consuming for large SNP panels (such as genome-wide association studies). Second, continuous variables have to be binned into categories to be considered for analysis, potentially leading to a loss of information. However, there are some approaches to alleviate this problem [8,9]. An alternative approach for the analysis of genetic association studies is the use of neural networks (NN) and their modifications, e.g. genetic programming neural networks (GPNN) [10] and grammatical evolution neural networks (GENN) [11]. If well trained, neural network models yield good predictivity and allow for the incorporation of continuous variables. However, neural network models are often perceived to have a "black box character" where deconvoluting the contribution and interaction between genetic markers can be challenging. A recent comparison of methods showed that each method demonstrates strengths and weaknesses and there is still a need for alternative methods, because no single method will perform well in all scenarios [4].

Thus, rule based classifier algorithms which have proven performance with non-genetic datasets [12], could provide a valuable complementary method for the analysis of genetic association studies. Rule based classifiers generate classification models using a collection of "if ... then ..." rules [12]. The algorithms are computationally inexpensive, are capable of incorporating categorical and continuous variables and the developed models are usually easy to interpret.

The aim of the current study was to evaluate the performance of three selected rule based classifier algorithms, RIPPER [13], RIDOR [14] and PART [15], for the analysis of genetic association studies. Simulated datasets with varying statistical power and three different case-control models were generated in order to perform these evaluations.

## Methods

### Algorithms

Three different deterministic rule based classifier algorithms, for which the mathematical background is extensively described in the literature, were evaluated in the current study: (a) RIPPER (Repeated Incremental Pruning to Produce Error Reduction) [13] (b) RIDOR (RIpple-DOwn Rule) [14] and (c) PART [15]. The RIPPER algorithm is a direct method, i.e. RIPPER extracts the rules directly from the data. The algorithm progresses through four phases: i) growth, ii) pruning, iii) optimization, iv) selection. In the growth phase, one rule is generated by greedily adding attributes to the rule until the rule meets stopping criteria. In the following prune phase, each rule is incrementally pruned, allowing the pruning of any final sequence of the attributes, until a pruning metric is fulfilled. In the optimization stage each generated rule is further optimized by a) greedily adding attributes to the original rule and b) by independently growing a new rule undergoing a growth and pruning phase, as described above. Finally, in the selection phase, the best rules are kept and the other rules are deleted from the model. RIDOR is also a direct method, first generating a default rule (e.g. "all patients are controls") and then exceptions ("except if rs5 = AB and rs10 = AB then patients are cases") to the default rule with the least error rate. The "best" exceptions for each exception are generated and iterated until pure. Thus, a tree-like expansion of exceptions is generated. The exceptions are a set of rules that predict classes other than the default. PART is an indirect method for rule generation. PART generates a pruned decision tree using the C4.5 statistical classifier [16] in each iteration. From the best tree, the leaves are translated into rules.

In the current study, the WEKA open-source software was used to implement the three rule-based classifier methods [17]. In WEKA, the RIPPER algorithm is implemented and named as JRIP (i.e. Java implementation of RIPPER).

### Datasets

For the evaluation of the algorithms, three different case-control models (A, B & C) (Table 1) were used for dataset simulation. Model A consisted of four case rules constructed by the interaction of two SNPs, rs5 and rs10. Model B consisted of two case rules constructed by the interaction of rs5 and rs10 and by the interaction between SNP rs15 and the area under the curve (AUC) of a hypothetical compound X, as an environmental factor. For model C cases were assigned randomly without any relationship to a genotype or environmental factor, i.e. a "null model".

**Table 1:** Case-Control Models

Model A	Model B	Model C
*Rule1:*	*Rule1*:	*Rule1:*
If rs5 = AA and rs10 = AB then Case	If rs5 = BB and rs10 = AA then Case	If random number > "threshold" then Case
*Rule2:*	*Rule2:*	*Rule 2:*
If rs5 = AB and rs10 = AA then Case	If rs15 = AA and AUC >105 then Case	Else Control
*Rule3:*	*Rule3:*	
If rs5 = AB and rs10 = BB then Case	Else Control	
*Rule4:*		
If rs5 = BB and rs10 = AB then Case		
*Rule 5:*		
Else Control		

The case-control models A, B and C were used to simulate in total 42 various datasets: the number of subjects (300, 600), the number of SNPs (500, 1500, 3000) and the noise of the case-control model with varying false positive (FP) and false negative (FN) rates (5%, 10%, 20%) was varied for models A and B resulting in 18 different datasets for each case-control model (A and B) and 6 different datasets for model C (Table 2). SNPs simulated in the datasets were named with a consecutive dbSNP [18] reference SNP identifier number (rs) from rs1 to rs1200. The reported rs numbers **do not**refer to their biological functionality as described in the dbSNP database [18]. For all other SNPs not used in the case-control models, no influence on the phenotype (i.e. case status) was assumed. The genotype frequencies were simulated randomly, uncorrelated and under assumption of Hardy-Weinberg Equilibrium (HWE) [19-21]. For the minor allele frequency (MAF) [22] a uniform distribution was selected ranging from 0 - 0.5 with randomly varying minor alleles. The environmental factor AUC was simulated by a uniform distribution with medians at 110 and 95 resulting in a binomial distribution of the AUC. For the simulation of each of the 42 datasets different random seeds were used. Dataset generation was performed using SAS (SAS Institute Inc., Cary, NC, V 9.1.3). Simulated datasets are available upon request.

**Table 2:** Datasets investigated

Dataset	Model	# SNPs	**#Patients**^**$**^	Ratio Control/Case	FP [%]	FN [%]
1	A	500	300	2	5	5
2	A	1500	300	2	5	5
3	A	3000	300	2	5	5
4	A	500	600	2	5	5
5	A	1500	600	2	5	5
6	A	3000	600	2	5	5
7	A	500	300	2	10	10
8	A	1500	300	2	10	10
9	A	3000	300	2	10	10
10	A	500	600	2	10	10
11	A	1500	600	2	10	10
12	A	3000	600	2	10	10
13	A	500	300	2	20	20
14	A	1500	300	2	20	20
15	A	3000	300	2	20	20
16	A	500	600	2	20	20
17	A	1500	600	2	20	20
18	A	3000	600	2	20	20

19	B	500	150+150	2	5	5
20	B	1500	150+150	2	5	5
21	B	3000	150+150	2	5	5
22	B	500	300+300	2	5	5
23	B	1500	300+300	2	5	5
24	B	3000	300+300	2	5	5
25	B	500	150+150	2	10	10
26	B	1500	150+150	2	10	10
27	B	3000	150+150	2	10	10
28	B	500	300+300	2	10	10
29	B	1500	300+300	2	10	10
30	B	3000	300+300	2	10	10
31	B	500	150+150	2	20	20
32	B	1500	150+150	2	20	20
33	B	3000	150+150	2	20	20
34	B	500	300+300	2	20	20
35	B	1500	300+300	2	20	20
36	B	3000	300+300	2	20	20

37	C	500	300	2	n.a.	n.a.
38	C	1500	300	2	n.a.	n.a.
39	C	3000	300	2	n.a.	n.a.
40	C	500	600	2	n.a.	n.a.
41	C	1500	600	2	n.a.	n.a.
42	C	3000	600	2	n.a.	n.a.

^$ ^For case rule A patients are equally distributed for the 4 case rules; for case rule B the first number indicates the number for rule 1, the second number for rule 2; n.a: not applicable.

### Data Analysis

Data analyses were performed using WEKA, version 3.7.0. For each algorithm, a varying set of algorithm specific options was applied resulting in 18, 30, and 9 different settings for RIPPER, PART and RIDOR, respectively (Table 3). The settings were chosen based on theoretical evaluations and based on previous experiences with the algorithms on similar datasets. Each of the settings (Table 3) was applied to each of the 42 simulated datasets (Table 2) using WEKA (command line mode). A customized Perl script was used to extract the most important information (e.g. file name, model, statistics, etc.) from the WEKA result files and to summarize the information in a comma separated file. A grading system (A - D) was created (Table 4), to further compare the results at three different levels: a) the Model level, b) the Rules level, and c) the Attribute level.

**Table 3:** Settings of Algorithm Options

Nr	RIPPER	RIDOR	PART
1	-F 3 -N 2.0 -O 10	-F 3 -S 1 -N 2.0 -A	-R -B -M 2 -N 3
2	-F 3 -N 5.0 -O 10	-F 3 -S 1 -N 5.0 -A	-R -B -M 5 -N 3
3	-F 3 -N 10.0 -O 10	-F 3 -S 1 -N 10.0 -A	-R -B -M 10 -N 3
4	-F 10 -N 2.0 -O 10	-F 10 -S 1 -N 2.0 -A	-R -B -M 2 -N 10
5	-F 10 -N 5.0 -O 10	-F 10 -S 1 -N 5.0 -A	-R -B -M 5 -N 10
6	-F 10 -N 10.0 -O 10	-F 10 -S 1 -N 10.0 -A	-R -B -M 10 -N 10
7	-F 100 -N 2.0 -O 10	-F 20 -S 1 -N 2.0 -A	-R -B -M 2 -N 100
8	-F 100 -N 5.0 -O 10	-F 20 -S 1 -N 5.0 -A	-R -B -M 5 -N 100
9	-F 100 -N 10.0 -O 10	-F 20 -S 1 -N 10.0 -A	-R -B -M 10 -N 100
10	-F 3 -N 2.0 -O 100		-R -M 2 -N 3
11	-F 3 -N 5.0 -O 100		-R -M 5 -N 3
12	-F 3 -N 10.0 -O 100		-R -M 10 -N 3
13	-F 10 -N 2.0 -O 100		-R -M 2 -N 10
14	-F 10 -N 5.0 -O 100		-R -M 5 -N 10
15	-F 10 -N 10.0 -O 100		-R -M 10 -N 10
16	-F 100 -N 2.0 -O 100		-R -M 2 -N 100
17	-F 100 -N 5.0 -O 100		-R -M 5 -N 100
18	-F 100 -N 10.0 -O 100		-R -M 10 -N 100
19			-B -M 2 -C 0.25
20			-B -M 2 -C 0.1
21			-B -M 5 -C 0.25
22			-B -M 5 -C 0.1
23			-B -M 10 -C 0.25
24			-B -M 10 -C 0.1
25			-M 2 -C 0.25
26			-M 2 -C 0.1
27			-M 5 -C 0.25
28			-M 5 -C 0.1
29			-M 10 -C 0.25
30			-M 10 -C 0.1

RIPPER: F: number of folds for reduced error pruning; N: minimal weights of instances within a split; O: number of optimization runs

RIDOR: F: number of folds for reduced error pruning; S: number of shuffles for randomization; A: Flag set to use the error rate of all the data to select the default class in each step. N: minimal weight of instances within a split.

PART: C: confidence threshold for pruning; M: minimum number of instances per leaf; R: use reduced error pruning; N: number of folds for reduced error pruning; B: Use binary splits for nominal attributes

**Table 4:** Grading System

Grade	Model	Rules	Attribute
A	100% accordance *Example:* *If rs5 = BB and rs10 = AA then Case; If rs15 = AA and AUC >105 then Case; Else Control*	100% accordance *Example:* *If rs5 = BB and rs10 = AA then Case*	All attributes were present (i.e. detected) and ranked as most frequent, i.e. top 2 for model A and top 4 for model B. *Example:* *rs5 *→ *Nr. 1; rs10 *→ *Nr. 3; rs15 *→ *Nr.2; AUC *→ *Nr. 4*

B	One attribute was missing or an additional attribute was identified by the generated model *Example:* *If rs5 = BB and rs10 = AA and rs234 = BB then Case; If rs15 = AA and AUC >105 then Case; Else Control*	One attribute was missing or an additional attribute was identified by the generated model *Example:* *If rs5 = BB and rs10 = AA and rs234 = BB then Case*	All attributes were present but not ranked as most frequent *Example:* *rs5 *→ *Nr. 5; rs10 *→ *Nr. 3; rs15 *→ *Nr.7; AUC *→ *Nr. 4*

C	Two attributes were different between the generated and the true model, *Example:* *If rs5 = BB and rs10 = AA and rs234 = BB then Case; If rs15 = AA and AUC >105 and rs56 = AA then Case; Else Control*	Two attributes were different between the generated and the true model *Example:* *If rs5 = BB and rs234 = BB then Case*	One attribute was not present, remaining attributes were present and rank was not considered *Example:* *rs5 *→ *not detected; rs10, rs15 and AUC *→ *detected*

D	Three attributes were different between the generated and the true model *Example:* *If rs5 = BB and rs234 = BB and rs56 = AA then Case; If rs15 = AA and AUC >105 then Case; Else Control*	Three attributes were different between the generated and the true model *Example:* *If rs5 = BB and rs56 = AA and rs234 = BB then Case*	Two attributes were not present, remaining attributes were present and ranked as most frequent *Example:* *rs5 and AUC *→ *not detected; rs10 and rs15 *→ *detected*

Comparison of the generated versus true model, rules and attributes. (Model B was used for the example.)

On the Model level, each developed model was compared to the true model. At the Rules level, each rule was extracted individually from each developed model and compared to the true case-control rule. At both levels, the "best" grade was always assigned to each model, where A is better than B, B better than C, etc.

On the Attribute level, all SNPs and other attributes used as case-control predictors were extracted from the respective models. The appearance of each marker was counted for each dataset and algorithm. The most frequent marker was ranked as 1^st^, the second most frequent marker was ranked as 2^nd^, and so forth. Subsequently, the grading system was applied (Table 4). If the attribute ranking fulfilled a grading specification, the dataset and algorithm was assigned the "best" grade, where A is better than B, B better than C, etc. For the evaluation of the "null model" (model C), the same procedure as described above was applied. The true model was assumed to be either model A and/or model B. For the rule and attribute evaluation, the respective rules and attributes from models A and/or B were applied.

## Results

In total, 2394 models (= 42 datasets × 57 algorithm options) were generated and analyzed as described in the methods section. A summary statistic on the number of rules per model and the number of attributes per model is presented in Table 5. A qualitative summary of the results is shown in Table 6. Detailed quantitative results at the Model, Rule and Attribute level are presented in Tables 7, 8, &9.

**Table 5:** Statistics

		RIPPER			RIDOR			PART		
		Model A	Model B	Model C	Model A	Model B	Model C	Model A	Model B	Model C
#	Min	1	3	1	2	3	1	1	1	2
Attributes* per model	5th Percentile	2	3	1	4	5	4	2	4	7
	Median	7	4	15	16	17	28	24	24	47
	95th Percentile	28	13	42	46	50	70	102	109	144
	Max	43	21	63	65	62	73	245	240	259

# rules^$ ^per model	Min	2	3	2	3	3	1	1	1	1
	5th Percentile	3	3	2	4	4	2	2	2	1
	Median	6	3	6	8	8	9.5	7	6	9
	95th Percentile	11	7	14	16	17	19	25.5	24.5	37.5
	Max	16	9	18	20	20	20	45	43	46

* unique SNP or unique environmental variable; ^$ ^Case and control rules combined

**Table 6:** Qualitative Results - Summary

		RIPPER			RIDOR			PART		
Evaluation Level	Grade	Model A	Model B	Model C	Model A	Model B	Model C	Model A	Model B	Model C
Models	A	33% (6)	56% (10)	0% (0)	0% (0)	11% (2)	0% (0)	0% (0)	33% (6)	0% (0)
	B	33% (6)	50% (9)	0% (0)	0% (0)	6% (1)	0% (0)	0% (0)	33% (6)	0% (0)
	C	22% (4)	56% (10)	0% (0)	0% (0)	6% (1)	0% (0)	0% (0)	22% (4)	0% (0)
	D	6% (1)	22% (4)	0% (0)	0% (0)	17% (3)	0% (0)	6% (1)	11% (2)	0% (0)

Rules	A	83% (15)	100% (18)	0% (0)	83% (15)	94% (17)	0% (0)	44% (8)	78% (14)	0% (0)
	B	89% (16)	72% (13)	0% (0)	94% (17)	100% (18)	0% (0)	50% (9)	100% (18)	0% (0)
	C	67% (12)	67% (12)	0% (0)	56% (10)	100% (18)	0% (0)	78% (14)	89% (16)	0% (0)
	D	50% (9)	56% (10)	0% (0)	67% (12)	100% (18)	0% (0)	56% (10)	94% (17)	0% (0)

Attributes	A	83% (15)	56% (10)	0% (0)	50% (9)	72% (13)	0% (0)	11% (2)	0% (0)	0% (0)
	B	17% (3)	28% (5)	0% (0)	50% (9)	17% (3)	0% (0)	61% (11)	78% (14)	0% (0)
	C	0% (0)	17% (3)	0% (0)	0% (0)	11% (2)	0% (0)	6% (1)	22% (4)	0% (0)
	D	0% (0)	0% (0)	0% (0)	0% (0)	0% (0)	0% (0)	22% (4)	0% (0)	0% (0)

Summary of the results, separated by algorithm, case-control model, and grading. Number represents the percent frequency of datasets where a respective grade was achieved at least once (absolute number is in brackets).

**Table 7:** Quantitative Results - Models Level

					RIPPER				RIDOR				PART			
Model	Dataset	SNPs	Patients	Error [%]	A	B	C	D	A	B	C	D	A	B	C	D
A	1	500	300	5	0	1	7	0	0	0	0	0	0	0	0	0
A	2	1500	300	5	0	0	0	0	0	0	0	0	0	0	0	2
A	3	3000	300	5	0	0	0	0	0	0	0	0	0	0	0	0
A	4	500	600	5	3	1	0	0	0	0	0	0	0	0	0	0
A	5	1500	600	5	7	5	0	0	0	0	0	0	0	0	0	0
A	6	3000	600	5	1	2	2	0	0	0	0	0	0	0	0	0
A	7	500	300	10	0	0	0	0	0	0	0	0	0	0	0	0
A	8	1500	300	10	0	1	0	0	0	0	0	0	0	0	0	0
A	9	3000	300	10	0	0	0	0	0	0	0	0	0	0	0	0
A	10	500	600	10	8	0	1	0	0	0	0	0	0	0	0	0
A	11	1500	600	10	0	0	0	7	0	0	0	0	0	0	0	0
A	12	3000	600	10	1	0	1	0	0	0	0	0	0	0	0	0
A	13	500	300	20	0	0	0	0	0	0	0	0	0	0	0	0
A	14	1500	300	20	0	0	0	0	0	0	0	0	0	0	0	0
A	15	3000	300	20	0	0	0	0	0	0	0	0	0	0	0	0
A	16	500	600	20	6	3	0	0	0	0	0	0	0	0	0	0
A	17	1500	600	20	0	0	0	0	0	0	0	0	0	0	0	0
A	18	3000	600	20	0	0	0	0	0	0	0	0	0	0	0	0

B	19	500	150+150	5	11	7	0	0	2	0	1	0	1	1	0	0
B	20	1500	150+150	5	15	0	3	0	0	1	0	0	2	0	0	0
B	21	3000	150+150	5	4	4	2	0	0	0	0	3	0	0	1	1
B	22	500	300+300	5	14	0	4	0	0	0	0	0	5	0	0	0
B	23	1500	300+300	5	18	0	0	0	0	0	0	0	0	2	0	0
B	24	3000	300+300	5	17	1	0	0	0	0	0	0	1	1	0	0
B	25	500	150+150	10	7	10	0	0	0	0	0	0	1	0	2	0
B	26	1500	150+150	10	0	2	2	0	0	0	0	1	0	1	2	1
B	27	3000	150+150	10	0	3	2	1	0	0	0	0	0	0	0	0
B	28	500	300+300	10	18	0	0	0	1	0	0	0	1	1	0	0
B	29	1500	300+300	10	17	1	0	0	0	0	0	0	0	1	1	0
B	30	3000	300+300	10	13	1	0	0	0	0	0	0	0	0	0	0
B	31	500	150+150	20	0	0	7	1	0	0	0	1	0	0	0	0
B	32	1500	150+150	20	0	0	1	0	0	0	0	0	0	0	0	0
B	33	3000	150+150	20	0	0	4	4	0	0	0	0	0	0	0	0
B	34	500	300+300	20	0	4	1	0	0	0	0	0	0	0	0	0
B	35	1500	300+300	20	0	0	1	0	0	0	0	0	0	0	0	0
B	36	3000	300+300	20	0	0	0	7	0	0	0	0	0	0	0	0

C	37	500	300	n.a.	0	0	0	0	0	0	0	0	0	0	0	0
C	38	1500	300	n.a.	0	0	0	0	0	0	0	0	0	0	0	0
C	39	3000	300	n.a.	0	0	0	0	0	0	0	0	0	0	0	0
C	40	500	600	n.a.	0	0	0	0	0	0	0	0	0	0	0	0
C	41	1500	600	n.a.	0	0	0	0	0	0	0	0	0	0	0	0
C	42	3000	600	n.a.	0	0	0	0	0	0	0	0	0	0	0	0

Summary of the results at the model level, separated by algorithm, case-control model, dataset and grading. Number expresses the absolute frequency of the respective grading assignment.

**Table 8:** Quantitative Results - Rules Level

					RIPPER				RIDOR				PART			
Model	Dataset	SNPs	Patients	Error [%]	A	B	C	D	A	B	C	D	A	B	C	D
A	1	500	300	5	44	14	3	0	4	6	2	3	12	13	2	2
A	2	1500	300	5	0	36	0	0	4	16	0	3	3	4	1	2
A	3	3000	300	5	0	1	7	0	0	1	0	0	0	0	0	0
A	4	500	600	5	46	14	0	0	30	20	0	0	25	28	27	10
A	5	1500	600	5	72	0	0	0	12	29	4	1	2	4	3	2
A	6	3000	600	5	28	32	0	0	8	27	0	0	12	6	2	1
A	7	500	300	10	23	27	4	7	2	5	0	1	2	3	2	0
A	8	1500	300	10	19	13	0	3	2	12	0	2	2	0	2	0
A	9	3000	300	10	1	11	12	11	0	1	3	3	0	0	0	0
A	10	500	600	10	46	16	0	0	11	23	7	0	25	20	11	2
A	11	1500	600	10	35	10	15	6	6	19	11	2	0	11	16	9
A	12	3000	600	10	48	5	20	0	7	7	0	0	0	0	3	1
A	13	500	300	20	11	21	8	3	1	4	3	6	0	0	4	2
A	14	1500	300	20	5	1	6	9	0	1	3	0	0	0	0	0
A	15	3000	300	20	3	0	1	0	1	0	0	7	0	0	0	0
A	16	500	600	20	48	17	7	1	8	6	17	4	0	2	1	0
A	17	1500	600	20	25	18	26	2	8	4	15	4	0	0	2	4
A	18	3000	600	20	0	19	1	3	1	6	2	4	0	0	1	0

B	19	500	150+150	5	29	4	3	0	8	13	5	5	31	17	9	2
B	20	1500	150+150	5	30	6	0	0	8	8	7	5	12	13	19	3
B	21	3000	150+150	5	27	5	13	8	7	5	11	7	4	21	8	8
B	22	500	300+300	5	36	0	0	0	8	35	9	3	41	41	15	10
B	23	1500	300+300	5	36	0	0	0	9	21	12	5	20	27	11	4
B	24	3000	300+300	5	35	1	0	0	9	19	10	10	13	22	25	8
B	25	500	150+150	10	25	11	1	1	4	4	17	20	12	20	14	1
B	26	1500	150+150	10	16	5	19	9	1	9	14	7	17	13	3	3
B	27	3000	150+150	10	18	4	11	14	0	14	3	9	0	5	1	1
B	28	500	300+300	10	36	0	0	0	17	8	9	8	28	18	14	10
B	29	1500	300+300	10	35	1	0	0	7	14	16	6	7	27	7	4
B	30	3000	300+300	10	31	7	2	0	7	9	12	9	2	17	14	7
B	31	500	150+150	20	18	0	13	10	4	3	9	5	0	1	3	2
B	32	1500	150+150	20	10	8	21	11	2	8	5	6	0	1	0	0
B	33	3000	150+150	20	9	7	18	2	1	2	7	9	0	1	0	1
B	34	500	300+300	20	18	17	3	13	4	5	18	13	2	1	6	1
B	35	1500	300+300	20	18	0	9	31	4	3	12	13	2	4	7	2
B	36	3000	300+300	20	16	2	23	20	5	3	5	8	1	3	1	1

C	37	500	300	n.a.	0	0	0	0	0	0	0	0	0	0	0	0
C	38	1500	300	n.a.	0	0	0	0	0	0	0	0	0	0	0	0
C	39	3000	300	n.a.	0	0	0	0	0	0	0	0	0	0	0	0
C	40	500	600	n.a.	0	0	0	0	0	0	0	0	0	0	0	0
C	41	1500	600	n.a.	0	0	0	0	0	0	0	0	0	0	0	0
C	42	3000	600	n.a.	0	0	0	0	0	0	0	0	0	0	0	0

Summary of the results at the rules level, separated by algorithm, case-control model, dataset and grading. Number expresses the absolute frequency of the respective grading assignment.

### Models Level Analyses

The models generated had in median 4 to 47 unique attributes per model (Table 5). The RIPPER algorithm utilized the fewest number of attributes (median: 4 to 15) whereas RIDOR and PART had a significantly higher number of attributes per model (median: 16 to 47). The number of attributes was approximately two times higher for the "null model" (model C) compared to models A and B. The number of rules per model was comparable between all three algorithms (median: 3 to 9.5).

The RIPPER algorithm performed well and was able to discover the true model (Grade A) at least once, 33% of the time with case-control model A and 56% of the time with case-control model B (Table 6). The RIDOR and PART algorithm performed poorly in model detection. Case-control model A was not discovered in any dataset by the two algorithms. Case-control model B was identified at least once in 11% and 33% of the datasets by RIDOR and PART, respectively.

When minor deviations from the true model were considered (Grades B - D), RIPPER was again able to detect significantly more models compared to the RIDOR and PART algorithms. Overall, case-control model B was better discovered by all three algorithms, compared to case-control model A. None of the methods detected for model C a false positive finding.

Table 7 provides a detailed summary of model level algorithm performance. In general, algorithms performed worse if the power of the dataset decreased, i.e. less subjects and/or more SNPs and/or higher FP/FN rates.

### Rules Level Analyses

At the rules level, the RIPPER algorithm performed well and was able to discover the true case rules (Grade A) at least once, 83% of the time with case-control model A and 100% of the time with case-control model B (Table 6). The RIDOR and PART algorithms also performed well in rule detection. For case-control model A, the true rules were discovered at least once 83% and 44% of the time with the RIDOR and PART algorithms, respectively. With case-control model B the RIDOR and PART algorithms were able to discover the true rule at least once in 94% and 78% of the datasets, respectively.

When deviations (Grades B - D) from the true case rules were allowed, all algorithms performed with high discovery rates. Overall the case rules of case-control model B were slightly better discovered by all three algorithms, compared to the case rules of case-control model A. None of the methods detected for the rules of model C a false positive finding.

Table 8 provides a detailed summary of rule level algorithm performance. All algorithms performed worse in rule detection if the power of the dataset decreased, i.e. less subjects and/or more SNPs and/or higher FP/FN rates.

### Attributes Level Analyses

At the attributes level, the RIPPER algorithm performed well. RIPPER yielded an "A" grade (all attributes present and ranked as the top 2 (model A) or the top 4 (model B) attributes) in 83% of the time with case-control model A and 56% of the time with case-control model B (Table 6). The RIDOR algorithm demonstrated satisfactory performance at the attribute level with 50% and 72% of the datasets yielding an "A" grade in case-control models A and B, respectively. The PART algorithm did not perform as well. With case-control model A, the grade "A" designation occurred only 11% of the time and with case-control model B, the "A" grade did not get assigned to any of the outputs. For model C, all grading (A-D) were zero across all methods.

Table 9 provides a detailed summary of attribute level algorithm performance. All algorithms performed worse in attribute detection, if the power of the dataset decreased, i.e. less subjects and/or more SNPs and/or higher FP/FN rates.

**Table 9:** Quantitative Results - Attributes Level

					RIPPER				RIDOR				PART			
Model	Dataset	SNPs	Patients	Error [%]	A	B	C	D	A	B	C	D	A	B	C	D
A	1	500	300	5	1	0	0	0	0	1	0	0	0	1	0	0
A	2	1500	300	5	1	0	0	0	1	0	0	0	0	1	0	0
A	3	3000	300	5	0	1	0	0	0	1	0	0	0	0	0	0
A	4	500	600	5	1	0	0	0	1	0	0	0	1	0	0	1
A	5	1500	600	5	1	0	0	0	1	0	0	0	0	1	0	0
A	6	3000	600	5	1	0	0	0	1	0	0	0	0	1	0	0
A	7	500	300	10	1	0	0	0	0	1	0	0	0	1	0	0
A	8	1500	300	10	1	0	0	0	1	0	0	0	0	1	0	0
A	9	3000	300	10	1	0	0	0	0	1	0	0	0	0	0	1
A	10	500	600	10	1	0	0	0	1	0	0	0	1	0	0	0
A	11	1500	600	10	1	0	0	0	1	0	0	0	0	1	0	0
A	12	3000	600	10	1	0	0	0	0	1	0	0	0	1	0	0
A	13	500	300	20	1	0	0	0	0	1	0	0	0	1	0	0
A	14	1500	300	20	0	1	0	0	0	1	0	0	0	0	0	1
A	15	3000	300	20	0	1	0	0	0	1	0	0	0	0	0	1
A	16	500	600	20	1	0	0	0	1	0	0	0	0	1	0	0
A	17	1500	600	20	1	0	0	0	1	0	0	0	0	1	0	0
A	18	3000	600	20	1	0	0	0	0	1	0	0	0	0	1	0

B	19	500	150+150	5	1	0	0	0	1	0	0	0	0	1	0	0
B	20	1500	150+150	5	1	0	0	0	1	0	0	0	0	1	0	0
B	21	3000	150+150	5	1	0	0	0	1	0	0	0	0	1	0	0
B	22	500	300+300	5	1	0	0	0	1	0	0	0	0	1	0	0
B	23	1500	300+300	5	1	0	0	0	1	0	0	0	0	1	0	0
B	24	3000	300+300	5	1	0	0	0	0	1	0	0	0	1	0	0
B	25	500	150+150	10	1	0	0	0	0	0	1	0	0	1	0	0
B	26	1500	150+150	10	0	1	0	0	1	0	0	0	0	1	0	0
B	27	3000	150+150	10	0	0	1	0	0	0	1	0	0	1	0	0
B	28	500	300+300	10	1	0	0	0	1	0	0	0	0	1	0	0
B	29	1500	300+300	10	1	0	0	0	1	0	0	0	0	1	0	0
B	30	3000	300+300	10	1	0	0	0	1	0	0	0	0	1	0	0
B	31	500	150+150	20	0	1	0	0	1	0	0	0	0	0	1	0
B	32	1500	150+150	20	0	0	1	0	0	1	0	0	0	0	1	0
B	33	3000	150+150	20	0	0	1	0	0	1	0	0	0	0	1	0
B	34	500	300+300	20	0	1	0	0	1	0	0	0	0	1	0	0
B	35	1500	300+300	20	0	1	0	0	1	0	0	0	0	1	0	0
B	36	3000	300+300	20	0	1	0	0	1	0	0	0	0	0	1	0

C	37	500	300	n.a.	0	0	0	0	0	0	0	0	0	0	0	0
C	38	1500	300	n.a.	0	0	0	0	0	0	0	0	0	0	0	0
C	39	3000	300	n.a.	0	0	0	0	0	0	0	0	0	0	0	0
C	40	500	600	n.a.	0	0	0	0	0	0	0	0	0	0	0	0
C	41	1500	600	n.a.	0	0	0	0	0	0	0	0	0	0	0	0
C	42	3000	600	n.a.	0	0	0	0	0	0	0	0	0	0	0	0

Summary of the results at the attribute level, separated by algorithm, case-control model, dataset and grading. A "1" reflects an affiliation to the respective grading, whereas a "0" reflects no affiliation.

## Discussion

At the model level, the RIPPER algorithm outperformed the other two evaluated algorithms. RIPPER discovered the true model (grade "A") or a slight variation of the true model (grade "B") >40% of the time with case-control model A and >70% of the time with case-control model B. The RIDOR and PART algorithms were not able to detect the true case-control model A. Even under less stringent conditions of evaluation (Grade B), allowing minor deviations from the true model, the two algorithms were not able to discover the true case-control model. For case-control model B the RIDOR and PART algorithms performed better, however, RIPPER still outperformed the two. In general, the RIDOR and PART algorithms tended to build overly complex prediction models with a median of 2 to 3 times more predictors compared to the RIPPER algorithm. This complexity was probably caused by the nature of the algorithm. The PART algorithm derives rules from decision trees and decision trees tend to build overly complex models [12]. With the RIDOR algorithm, the "exception from the exception" principle is utilized for data investigation, this may not be appropriate to analyze complex gene-gene or gene-environment interactions.

All three algorithms performed well in detecting case-control rules. As with model level analyses, the RIPPER algorithm was superior to the RIDOR and PART algorithms in rule level analyses. The RIPPER algorithm discovered rules at a much higher frequency, even when corrected for the number of options tested (Table 3). If the frequency of each rule is counted and the rules ordered according to their frequencies (analogous to the attribute level analyses), the RIPPER algorithm identified more top-ranked rules than the other two algorithms.

At the attribute level, the RIPPER algorithm performed slightly better than RIDOR. The RIPPER algorithm discovered and top-ranked the true attributes 83% and 56% of the time for case-control models A and B, respectively. In contrast, the RIDOR algorithm discovered and top-ranked the true attributes 50% and 72% of the time for case-control models A and B, respectively. The PART algorithm performed the worst of the three algorithms but still identified the true attributes 72% and 78% of the time for case-control models A and B, respectively. The PART algorithm did not rank the true attributes in the top 2 (model A) or 4 (model B) as consistently as the RIPPER and RIDOR algorithms.

In addition to the two case-control models A and B datasets without relationships between genotypes and phenotypes (model C, "null model") were also tested. The null model results were not useful in further discriminating between the three rule based classifiers. For all datasets and with all methods, no false positive number of models, attributes or rules were identified. Nevertheless, the models derived from the "null model" datasets showed that the median number of attributes per model was twice as high compared to the real case-control models A and B. The number of attributes per model might therefore be a mechanism to discriminate true models from false positive models.

The RIPPER algorithm appears to be superior to the PART and RIDOR algorithm at all levels: models, rules and attributes. The tested set of algorithm options (Table 3) provides an adequate toolset for comparison; however, this work could be expanded and further optimized using simulated and non-simulated data.

Based on the analysis presented, questions may arise on how to best translate the rule based classifier into practice and how to apply the classifiers to non-simulated data. In data analysis practice, one would apply a selected classifier, preferably RIPPER, to one dataset with a battery of options, e.g. as provided in Table 3. Thus, for RIPPER this would result in 18 different models, one for each setting. For each of the 18 models standard statistics, e.g. numbers of rules, sensitivity, specificity, accuracy, etc., are provided. The next steps would be mainly triggered by the purpose of the analysis, depending on whether the analyst aims to use the classifiers as model builders or as filters. If the classifier is to be used for model building, the revealed models should be further investigated, e.g. by thorough review considering statistics such as sensitivity, specificity and complexity of the models. If classifiers are to be used as filters for attributes, similar procedures as described in the methods section could be applied. Additional research is required regarding practical considerations and evaluation methods, such as cross validation or external prediction.

In the presented analyses, the size of the datasets tested was limited to 3000 SNPs and 600 patients. Technically, rule based classifiers are neither limited by number of SNPs nor by number of patients. Thus, the methods should be scalable to whole genome levels. However, the size of the dataset analyzed in WEKA may be limited by the available computational memory and has to be taken into account.

## Conclusions

The current analyses substantiate the utility of rule based classifiers such as RIPPER, RIDOR and PART for the detection of gene-gene and gene-environment interactions in genetic association studies. These methods could provide a valuable new method, complementing existing approaches, in the analysis of genetic association studies. The methods provide an advantage in being able to handle both categorical and continuous variable types, and since the outputs of the analyses are easy to interpret the rule based classifier approach could quickly generate testable hypotheses for further evaluation. In addition, since the algorithms are computationally inexpensive to run, they may serve as valuable tools for preselection of attributes to be used in more complex, computationally intensive approaches such as MDR. Whether used in isolation or in conjunction with other tools, rule based classifiers are an important addition to the armamentarium of tools available for analyses of complex genetic association studies. As a next step, the most promising algorithm RIPPER should be benchmarked against other popular analysis methods, such as MDR or Random Forests.

## Competing interests

TL, DZ, JY, SJ and MDR have no competing interests.

## Authors' contributions

TL designed the analysis, established the algorithms, performed the analysis and drafted the manuscript. JY contributed to the design of the simulated data and the scientific framing of queries to be performed. DZ developed the Perl summary scripts and contributed to the design of the analysis. SJ contributed to the establishment of the algorithms and the manuscript. MDR participated in the design and coordination of the analysis and helped to draft the manuscript. All authors read and approved the final manuscript.
